# Microscopic and endoscopic “chopstick” technique removal of choroid plexus papilloma in the third ventricle of an infant: a case report with systematic review of literature

**DOI:** 10.3389/fonc.2023.1182261

**Published:** 2023-06-26

**Authors:** Weiwei Li, Zongping Li, Chengyan Wei, Xiaoyong Yang, Yuzhu Ji, Hongyuan Liu

**Affiliations:** ^1^ Department of Neurosurgery, Jiange People’s Hospital, Jiange, Sichuan, China; ^2^ Department of Neurosurgery, Mianyang Central Hospital, School of Medicine, University of Electronic Science and Technology of China, Mianyang, Sichuan, China; ^3^ Department of Pathology, Mianyang Central Hospital, School of Medicine, University of Electronic Science and Technology of China, Mianyang, Sichuan, China

**Keywords:** microscopic, “chopstick” technique, choroid plexus papilloma, infant, endoscopic

## Abstract

**Background:**

Choroid plexus papilloma (CPP) is rare and even rarer in infants and young children, and it usually occurs in the ventricles. Due to the physical peculiarities of infants, tumor removal by microscopic or endoscopic surgery alone is difficult.

**Case Presentation:**

A 3-month-old patient was found to have an abnormally enlarged head circumference for 7 days. Cranial magnetic resonance imaging (MRI) examination revealed a lesion in the third ventricle. The patient underwent combined microscopic and endoscopic “chopstick” technique to remove the tumor. He recovered well after the surgery. Postoperative pathological examination revealed CPP. Postoperative MRI suggested total resection of the tumor. Follow-up for 1 month showed no recurrence or distant metastasis.

**Conclusions:**

Combined microscopic and endoscopic “chopstick” technique may be a suitable approach to remove tumors in infant ventricles.

## Introduction

Choroid plexus papilloma (CPP) is a neuroectodermal tumor that accounts for less than 1% of intracranial tumors (1). CPP is even less frequent in infants. CPP is most often located in the lateral ventricles, with few reported cases in the third and fourth ventricles (2). Typical clinical manifestations of CPP include hydrocephalus, macrocephaly, and growth retardation (3, 4). The most common treatment for CPP is surgery. Transcallosal or transcortical approach are used to remove CPP located in the lateral ventricles. The corpus callosum approach is usually used to remove CPP located in the third ventricle. The blood supply of CPP is rich, and CPP is poorly demarcated from the surrounding tissues, which makes it difficult to remove the tumor with gross total resection. In particular, low tolerance to blood loss in infants makes surgical removal of CPP more difficult. With the advances in neurosurgical microscopy and improvement in endoscopic techniques, it has become possible for neurosurgeons to remove huge tumors from the ventricles minimally-invasively and completely. The resection of ventricular tumor was performed using microscopy or traditional endoscopic techniques. Here, we report a case of a 3-month-old infant with CPP in the third ventricle that was treated by combined microscopic and “chopstick” technique. We also conducted a systematic review of cases with CPP in infants reported after 2010.

## Case presentation

**Figure 1:**
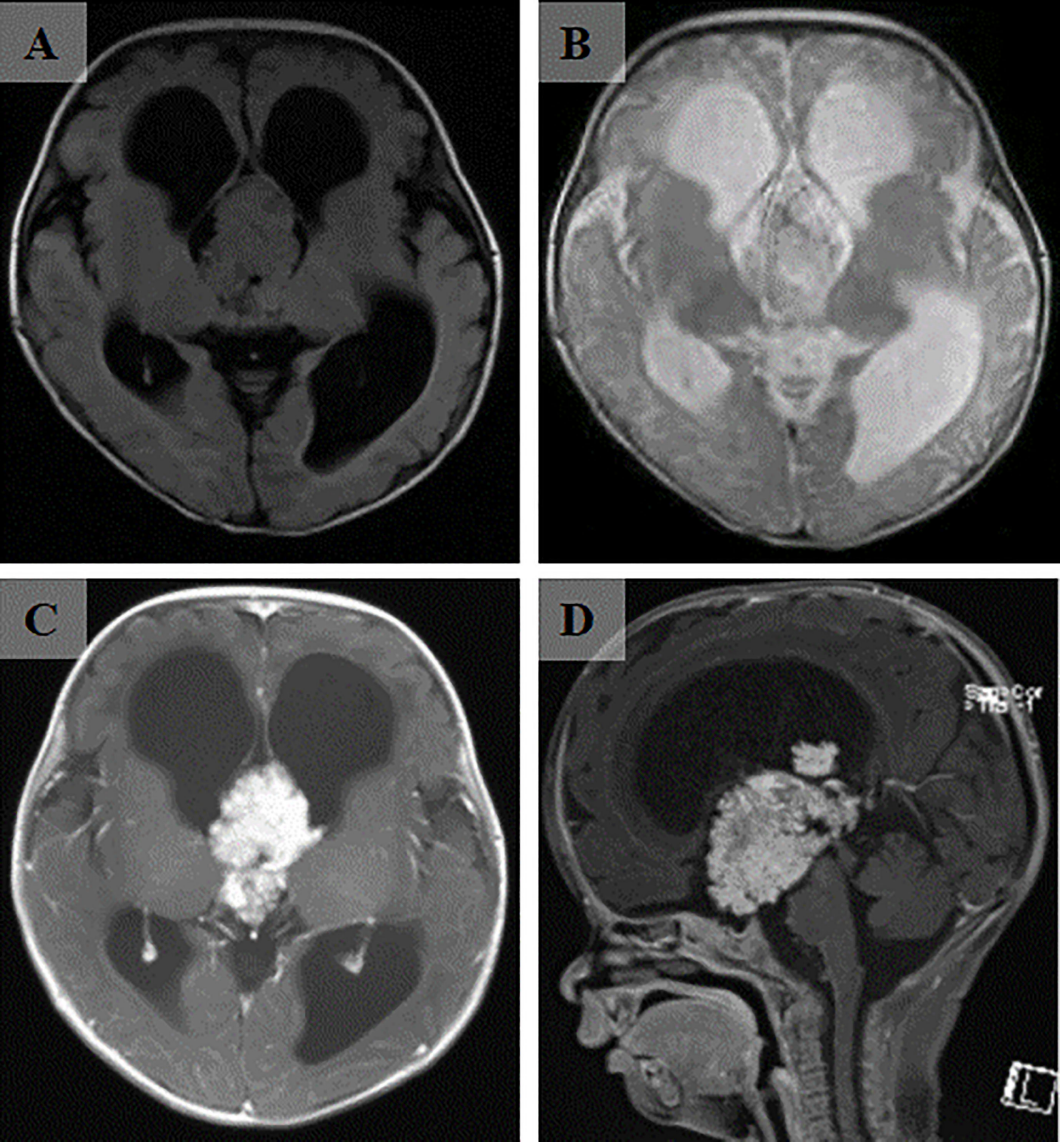
The cranial MRI of the head revealed a massive mass in the third ventricle. **(A)** The lesion was isointense on T1-weighted image. **(B)** The lesion was hyperintense on T2-weighted image. **(C)** After enhancement injection, the lesion in the third ventricle was significantly enhanced. **(D)** Sagittal view showed a “cauliflower-shaped” lesion after enhancement.

**Figure 2:**
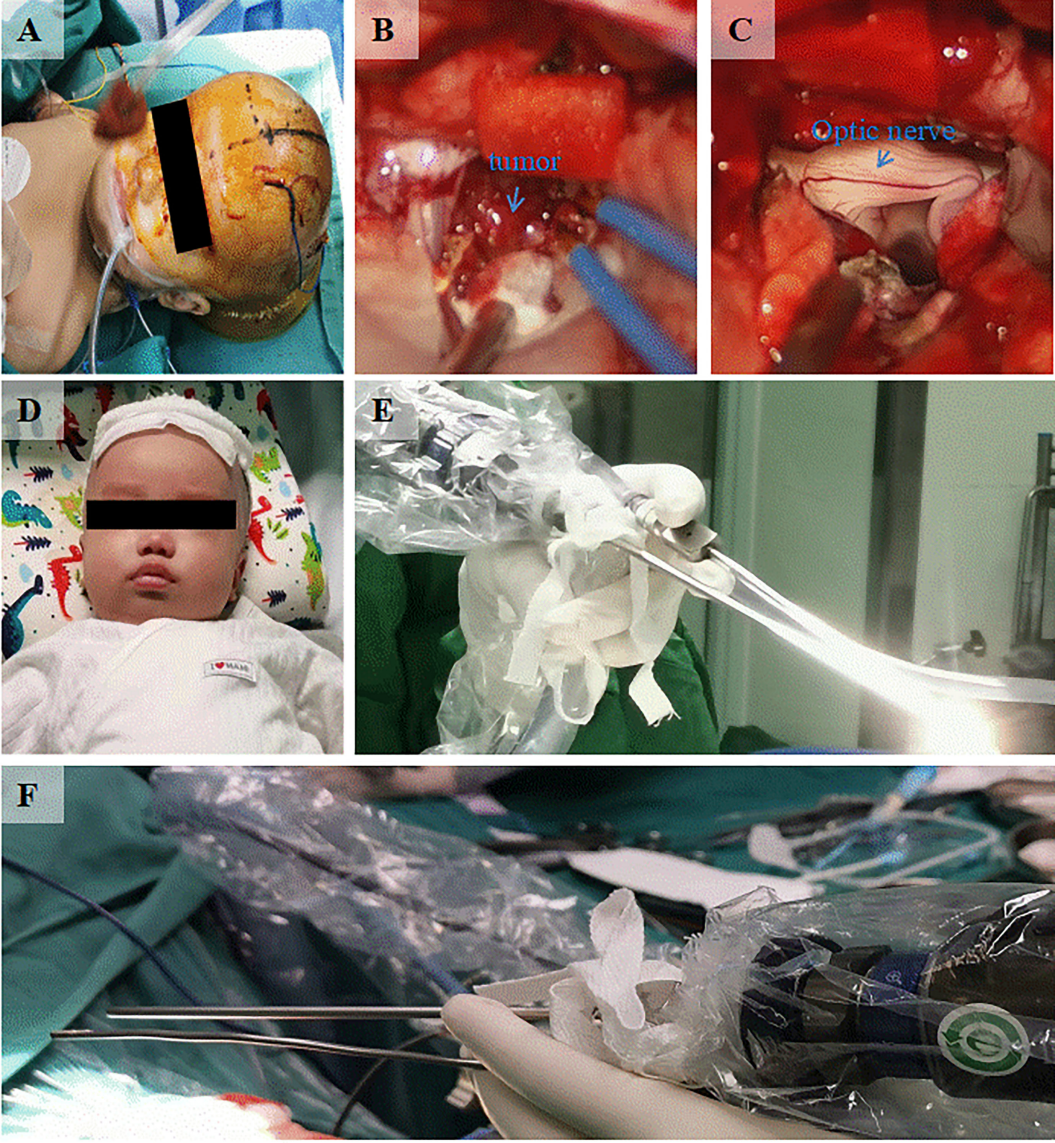
Intraoperative and postoperative conditions. **(A)** Appearance of the head during the surgical treatment. **(B)** Microscopic resection of the tumor by right frontal cortex fistula. **(C)** Exposure of optic nerve revealed after the tumor resection by microscope and endoscopic “chopstick” technique. **(D)** The recovery of the infant before discharge from the hospital. **(E)** Front view of the endoscopic “chopstick” technique. **(F)** Behind view of the endoscopic “chopstick” technique.

**Figure 3:**
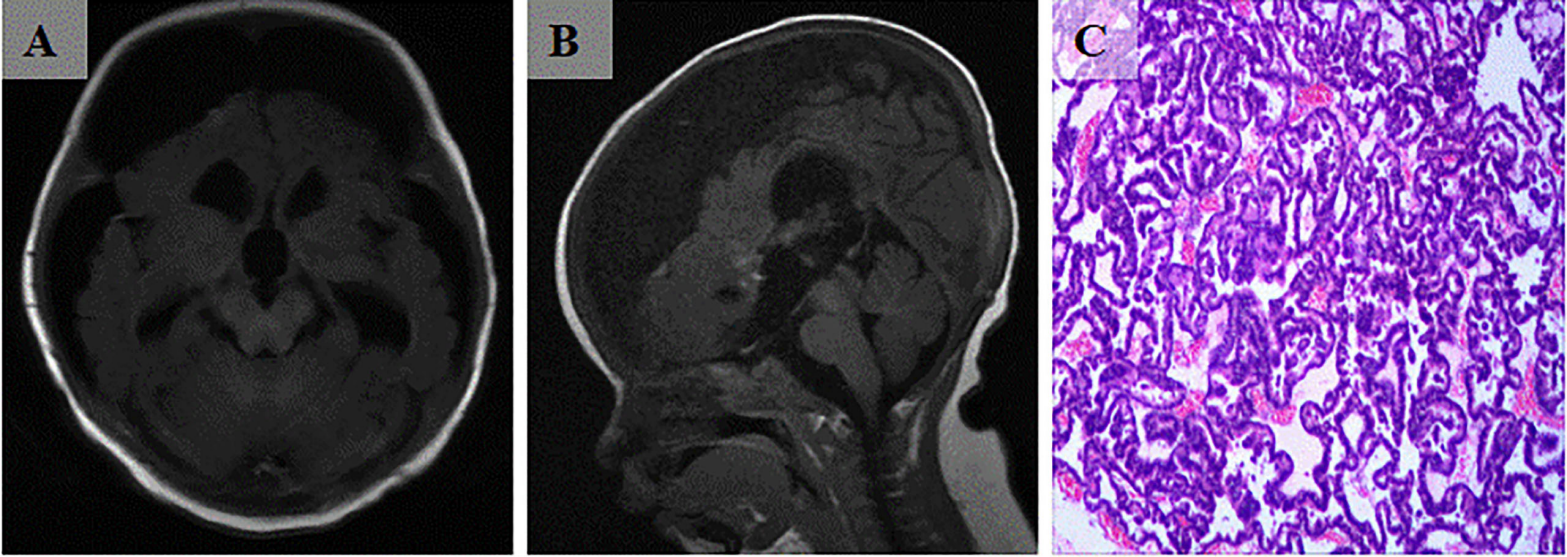
**(A, B)** Postoperative cranial MRI scan indicates total tumor resection. **(C)** Micrograph shows tumor papillary-like architecture that has fibrovascular cores (H and E, ×200).

A 3-month-old patient presented with an abnormally large head circumference for 7 days. He was born vaginally at full term, without asphyxia or birth injuries, and was breastfed after birth. Growth and intelligence were appropriate for his age. On examination, the fontanelle pressure was high, the head circumference was 45 cm, and the eyes were gazing downward. Ultrasound examination performed at other hospitals suggested hydrocephalus. Cranial magnetic resonance imaging (MRI) showed the presence of a space-occupying lesion in the third ventricle, about 3.8×4.6×3.1 cm (3) in size, isosignal at T1 and mild high signal at T2-weighted images, accompanied with severe hydrocephalus (**Figures 1A, B**). Contrast-enhanced lesions showed “cauliflower-like” appearance on MRI (**Figures 1C, D**). Right frontal cortical fistula was performed to remove a part of the tumor under the microscope, and the tumor in the blind area of the microscope’s visual field was removed by the endoscopic “chopstick” technique (**Figures 2A-C, E, F**). The child recovered well after surgery (**Figure 2D**).Histopathological examination of the resected tumor papillary-like architecture that has fibrovascular cores, resulting in a diagnosis of CPP (WHO Grade 1) (**Figure 3C**). After 1 month of follow-up, MRI indicated no residual tumor (**Figures 3A, B**).

## Systematic review of literature

Our literature search was based on the Preferred Reporting Items for Systematic Evaluation and Meta-Analysis (PRISMA) (5). We systematically searched reports in the English language from PubMed and EMBASE databases since 2010 using the Boolean operators with the following keywords: “Choroid plexus papilloma,” “infant,” “baby,” “neonatal,” and “young.” Articles with sufficient information for postoperative pathological diagnosis of CPP in individuals under the age of 12 months were included. Articles that replicated, reviewed, or lacked detailed clinical data were excluded. Cases with postoperative pathological diagnoses of atypical CPP (aCPP) and choroid plexus carcinoma (CPC) were excluded. All identified articles were first screened by title and abstract, and then the full text was downloaded and assessed for eligibility. This process was carried out independently by three investigators. Any disagreements were resolved by consensus.

## Results

**Figure 4:**
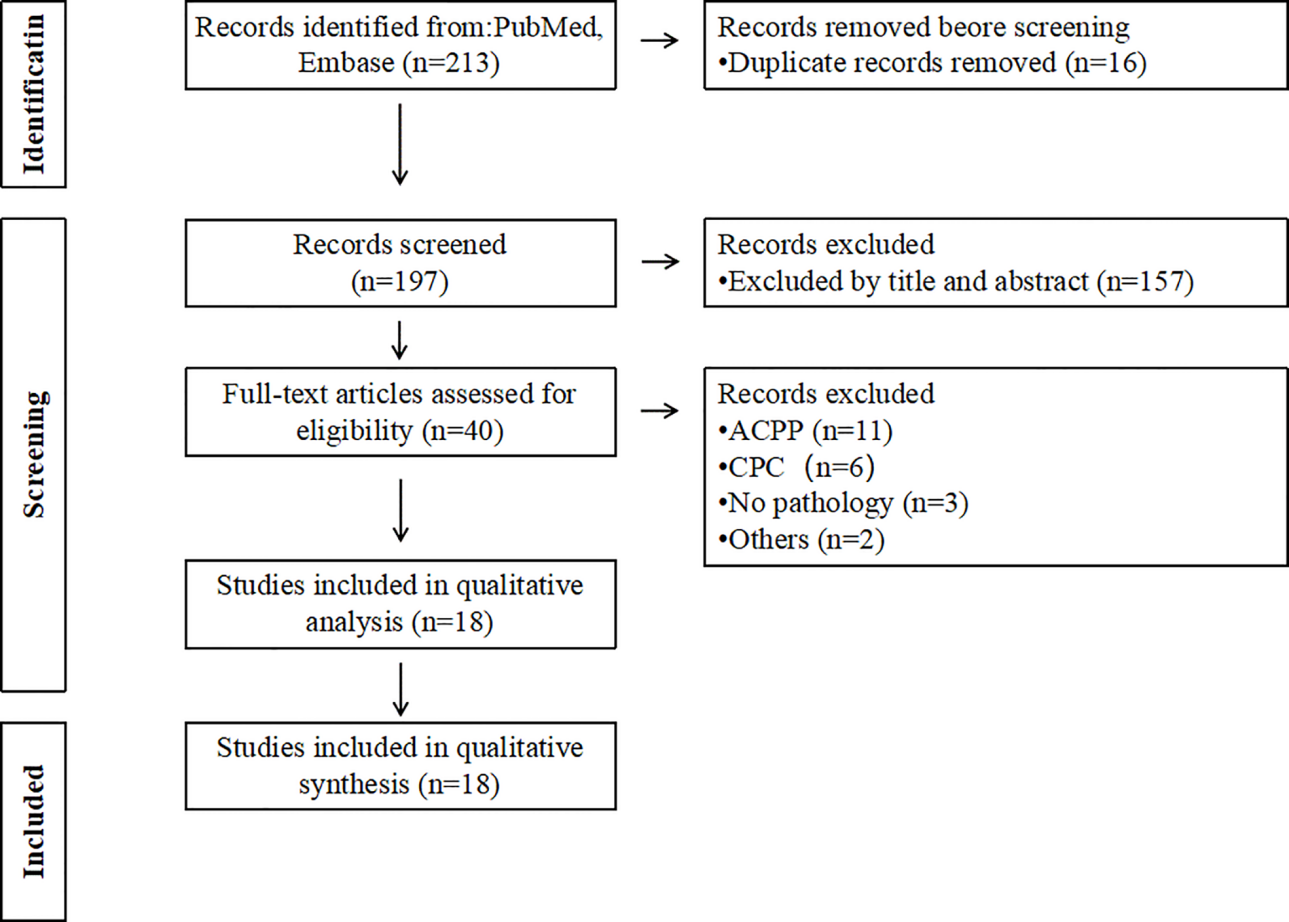
Flowchart outlining literature search using PRISMA.

Sixteen duplicate records were removed by database search. A total of 197 records were left for title and abstract checking. Forty full-text articles were evaluated. Eighteen articles were finally included for analysis (**Table 1**; **Figure 4**) (1, 3, 4, 6–20). The majority of the articles were case reports, and included a total of 30 cases with a pathological diagnosis of CPP. There were 18 males and nine females among the 30 patients, and sex was unknown in three cases. The minimum age was 3 days. The clinical manifestations of CPP were mainly enlarged head circumference (n=13) and high intracranial pressure (n=10). Twenty-two CPP cases were located in the lateral ventricle, while eight cases were located in the third ventricle. All 30 children were treated surgically, including 21 with microscopic resection alone, three with endoscopic resection alone, two with interventional embolization followed by microscopic resection, one with combined traditional endoscopic and microscopic treatment followed by cerebrospinal fluid shunt, and three with unspecified surgical modalities. The use of microscopy combined with the endoscopic “chopstick” technique had not been reported.

**Table 1:** Summary of the included studies.

Author and Year	No. of Patients	Age/Average Age(m)	Sex	Study Design	Symptoms	Tumor Site	Conclusions
Zhou et al., 2022 (1)	1	5	F	Case Report	Seizures	Lateral ventricle	Epileptic spasm syndrome as an initial clinical manifestation of CPP in pediatric patients without hydrocephalus is extremely rare.
Mangham et al., 2022 (4)	1	3	NA	Case Report	Lethargy	Third ventricle	CPP embolization may represent a curative strategy or facilitate delayed surgical resection in cases.
Puerta Roldán et al., 2019 (6)	1	4	M	Case Report	Increasing head size	Lateral ventricle	Diffuse enhanced pia may resolve spontaneously after CPP is removed. Many aspects should be considered in the treatment of CPP to avoid unnecessary treatment.
Misiolek et al., 2019 (3)	1	3	F	Case Report	Seizures	Lateral ventricle	The presence of multifocal CPP in children suggests a genetic predisposition. Rapid progression does not necessarily indicate a malignant tumor.
Dash et al., 2019 (7)	7	6.7	F (n=2), M (n=5)	Retrospective Case Series	Increasing head size (n=2), high intracranial pressure (n=5)	Lateral ventricle	CPP is associated with lesser blood loss and favorable outcome compared with aCPP and CPC.
Cao et al., 2018 (8)	1	4	F	Case Report	Increasing head size	Lateral ventricle	Total surgical removal is a valid curative method for CPP.
Laarakker et al., 2017 (9)	1	11	F	Case Report	Accidental discovery	Lateral ventricle	Asymptomatic CPP patients are uncommon, and treatment plans need to be tailored on a case-by-case basis.
Aljared et al., 2016 (10)	1	0.1	M	Case Report	High intracranial pressure	Lateral ventricle	Preoperative embolization of CPP allows complete resection and survival of this potentially curable disease.
Pandey et al., 2016 (11)	1	9	F	Case Report	Seizures	Lateral ventricle	Timely surgical intervention and complete resection of CPP with efforts to reduce intraoperative bleeding is the best treatment strategy.
Sufianov et al., 2015 (12)	1	5	M	Case Report	Increasing head size	third ventricle	Neuroendoscopy seems to be very promising in the treatment of children with intraventricular lesions.
Santos et al., 2015 (13)	1	2.5	M	Case Report	Increasing head size	Third ventricle	Endoscopic surgery can be an additional tool to consider when planning a choroid plexus tumor treatment approach.
Kennedy et al., 2015 (14)	5	4.2	F (n=2), M (n=3)	Retrospective Case Series	Increasing head size (n=2), high intracranial pressure (n=2), prenatal diagnosis (n=1)	Lateral ventricle	The superior parietal lobule approach is safe and effective for young children with CPP in the lateral ventricle. Preoperative embolization is not essential to avoid transfusion or achieve overall good outcomes.
Mizowaki et al., 2014 (15)	2	6.5	M	Case Report	Increasing head size, personality change	Third ventricle	Preservation of the venous system and ligation of the supplying arteries are critical before removal of CPP in young children.
Phi et al., 2014 (16)	2	3.5	NA	Case Report	Increasing head size	Lateral ventricle	Tranexamic acid may reduce intraoperative blood loss in infants with CPP.
Gupta et al., 2013 (17)	1	1.75	M	Case Report	Increasing head size	Third ventricle	CPP in the third ventricle is a rare infantile brain tumor.
Lysyy et al., 2012 (18)	1	6	M	Case Report	Increasing head size	Lateral ventricle	In newborns and young children, ultrasound is usually the first choice for the evaluation of bulging fontanelle or abnormal head circumference.
Reddy et al., 2011 (19)	1	1.5	M	Case Report	High intracranial pressure	Third ventricle	Third ventricular CPP in an infant was successfully resected using staged endoscopic and microsurgical approach.
Phi et al., 2011 (20)	1	7	M	Case Report	High intracranial pressure	Third ventricle	Excessive drainage of CSF combined with infusion of hypotonic saline with a physiologic potassium supplement can cause a catastrophic electrolyte imbalance in small infants with CPP.

CPP, Choroid plexus papilloma; F, female; M, male; m, month; aCPP, atypical choroid plexus papilloma; CPC, choroid plexus carcinoma; CSF, Cerebrospinal fluid.

## Discussion

CPP is a rare intracranial tumor that is classified as grade I by the WHO tumor classification. In pediatric patients, CPP usually occurs within 1 year of birth and is most commonly located in the lateral ventricle (64%) (21). According to our literature review, under the age of 1 year, CPP was located in the lateral ventricle in 73.3% of cases. Given that the location of CPP affects the cerebrospinal fluid circulation, hydrocephalus is often complicated in imaging. On computed tomography (CT) scans, CPP usually appears as isodense or hyperdense. MRI shows equal or low T1 signal and high T2 signal (2). In addition to surgical removal of the tumor, cerebrospinal fluid shunts or interventional embolization can be performed preoperatively (10). The most common surgical treatment for CPP is transcortical approach by a microscope (22). The survival rate after surgical resection of CPP is high, and it has been reported that the long-term survival rate ranges from 90% to 100% with gross total resection (11). For infant patients with a high surgical risk, preoperative tumor interventional embolization has been proposed (4, 10). Preparation of cerebrospinal fluid shunt alone may also complement CPP treatment (19). Because lesions are often located within the ventricle, microscope alone has a limited field of vision, so endoscopy can be used as a surgical supplement. Simple endoscopic resection of ventricular lesions alone is commonly used for tumors of smaller size. The endoscopic system is demanding for the operator and often requires skilled coordination and cooperation between the primary surgeon and assistant, and poor coordination may even increase the risk of additional procedures for the patient. Manickavasagam proposed the “chopstick” technique in 2010 (23). Labidi further elaborated the application of “chopstick” technique in 2018, in which the operator holds the endoscope and a suction device in one hand, while the other hand holds the instrument to perform various operations (24). This technique reduces the space required for surgery, alleviates the disadvantages of unstable holding of the mirror by the assistant for complex surgery, and avoids uncoordinated operation between the operator and assistant (24).

In our report, the lesion was first resected using the microscopic access through the frontal cortex, and then the tumor in the blind area of the microscope’s visual field was removed by endoscopic “chopstick” technique to achieve complete resection. In the “chopstick” technique, the endoscope and suction device are held in the left hand. The light source connector of the endoscope is placed between the thumb and ring fingers, and the direction can be changed as required during the operation. The suction device is placed between the index and middle fingers. A 0° or 30° endoscope can be used depending on the location of the lesion. This provides a new method for total resection of ventricle tumors with minimal trauma. Clearly, as this is a case report only, more studies with appropriate design and larger size are needed to provide stronger evidence level.

## Conclusions

Microscope combined with endoscopic “chopstick” technique may be a new way to remove the tumors in the ventricle.

## Data availability statement

The original contributions presented in the study are included in the article/supplementary material. Further inquiries can be directed to the corresponding author.

## Author contributions

Investigation: WL, CW, XY. Methodology: HL, ZL. Project administration: HL, WL. Resources: HL, ZL. Supervision: HL. Histopathological examination: YJ. All authors contributed to the article and approved the submitted version.

## References

[1] Zhou F Li Y Shen L Yao H Hou X . Infantile epileptic spasms syndrome as an initial presentation in infantile choroid plexus papilloma: a case report. Front Pediatr (2022) 10:1035621. doi: 10.3389/fped.2022.1035621 36467493 PMC9709204

[2] Prasad GL Mahapatra AK . Case series of choroid plexus papilloma in children at uncommon locations and review of the literature. Surg Neurol Int (2015) 6:151. doi: 10.4103/2152-7806.166167 26500797 PMC4596056

[3] Misiolek KA Osborn ZG Hauser N Thomas D Goodman JF Fulkerson DH . Rapidly growing, multifocal, benign choroid plexus tumor in an infant: case report. J Neurosurg Pediatr (2019) 22:1. doi: 10.3171/2018.12.PEDS18453 30797210

[4] Mangham WM Elijovich L Lee-Diaz JA Orr BA Gienapp AJ Boop FA . Pre-operative embolization for staged treatment of infantile choroid plexus papilloma. Child’s nervous system (2022) 38(2):429–33. doi: 10.1007/s00381-021-05212-w 34009420

[5] Preferred Reporting Items for Systematic Reviews and Meta-Analyses (PRISMA) . Flow diagram template (2020). Available at: https://journalsplosorg/plosmedicine/article/figure?id=101371/journalpmed1003583g001.

[6] Puerta Roldán P Santa-María López V Morales La Madrid A Cruz O Muchart J Thomas C . Vanishing diffuse leptomeningeal contrast enhancement in an infant with choroid plexus papilloma. Acta neurochirurgica (2019) 161(2):351–4. doi: 10.1007/s00701-018-03781-5 30617713

[7] Dash C Moorthy S Garg K Singh PK Kumar A Gurjar H . Management of choroid plexus tumors in infants and young children up to 4 years of age: an institutional experience. World Neurosurg (2019) 121:e237–45. doi: 10.1016/j.wneu.2018.09.089 30261376

[8] Cao LR Chen J Zhang RP Hu XL Fang YL Cai CQ . Choroid plexus papilloma of bilateral lateral ventricle in an infant conceived by *in vitro* fertilization. Pediatr Neurosurg (2018) 53(6):401–6. doi: 10.1159/000491639 30391955

[9] Laarakker AS Nakhla J Kobets A Abbott R . Incidental choroid plexus papilloma in a child: a difficult decision. Surg Neurol Int (2017) 8:86. doi: 10.4103/sni.sni_386_16 28607820 PMC5461574

[10] Aljared T Farmer JP Tampieri D . Feasibility and value of preoperative embolization of a congenital choroid plexus tumour in the premature infant: an illustrative case report with technical details. Interventional neuroradiol (2016) 22(6):732–5. doi: 10.1177/1591019916665346 PMC5564368 27605545

[11] Pandey S Sharma V Singh K Ghosh A Gupta PK . Uncommon presentation of choroid plexus papilloma in an infant. J Pediatr Neurosci (2016) 11(1):61–3. doi: 10.4103/1817-1745.181254 PMC4862292 27195037

[12] Sufianov AA Gaibov SS Sufianov RA . Endoscopic monoportal removal of a choroid plexus papilloma in the posterior third ventricle in a child. J Neurosurg Pediatr (2015) 16(1):107–11. doi: 10.3171/2014.12.PEDS14306 25910036

[13] Santos MM Souweidane MM . Purely endoscopic resection of a choroid plexus papilloma of the third ventricle: case report. J Neurosurg Pediatr (2015) 16(1):54–7. doi: 10.3171/2014.12.PEDS14287 25860986

[14] Kennedy BC Cloney MB Anderson RC Feldstein NA . Superior parietal lobule approach for choroid plexus papillomas without preoperative embolization in very young children. J Neurosurg Pediatr (2015) 16(1):101–6. doi: 10.3171/2014.11.PEDS14281 25860983

[15] Mizowaki T Nagashima T Yamamoto K Kawamura A Yoshida M Kohmura E . Optimized surgical approach to third ventricular choroid plexus papillomas of young children based on anatomical variations. World Neurosurg (2014) 82(5):912.e15–9. doi: 10.1016/j.wneu.2013.03.011 23510722

[16] Phi JH Goobie SM Hong KH Dholakia A Smith ER . Use of tranexamic acid in infants undergoing choroid plexus papilloma surgery: a report of two cases. Paediatric anaesthesia (2014) 24(7):791–3. doi: 10.1111/pan.12447 24924340

[17] Gupta P Sodhi KS Mohindra S Saxena AK Das A Khandelwal N . Choroid plexus papilloma of the third ventricle: a rare infantile brain tumor. J Pediatr Neurosci (2013) 8(3):247–9. doi: 10.4103/1817-1745.123696 PMC3888048 24470825

[18] Lysyy O Puzhevsky A Strauss S . Choroid plexus papilloma in an infant: ultrasound diagnosis. Eur J Pediatr (2012) 171(11):1717–8. doi: 10.1007/s00431-012-1842-1 23015045

[19] Reddy D Gunnarsson T Scheinemann K Provias JP Singh SK . Combined staged endoscopic and microsurgical approach of a third ventricular choroid plexus papilloma in an infant. Minimally invasive neurosurgery: MIN (2011) 54(5-6):264–7. doi: 10.1055/s-0031-1287775 22278793

[20] Phi JH Shin CH Wang KC Park SH Kim SK . Catastrophic electrolyte imbalance caused by excessive production and overdrainage of cerebrospinal fluid in an infant with choroid plexus papilloma. Child’s nervous system (2011) 27(7):1153–6. doi: 10.1007/s00381-011-1459-0 21503754

[21] Basindwah SA Alzahrani BS Ajlan AM Alkhalidi H . Persistence of communicating hydrocephalus post choroid plexus tumor resection: case reports and review of literature. Surg Neurol Int (2021) 12:483. doi: 10.25259/SNI_681_2021 34754533 PMC8571326

[22] Malomo TA Okolo CA Balogun JA . Endoscopic assisted, transfontanelle excision of a Large third ventricular atypical choroid plexus papilloma in an infant. J West Afr Coll Surgeons (2018) 8(4):136–50. PMC7861192 33553056

[23] Manickavasagam J Segaram S Harkness P . Functional endoscopic sinus surgery chopstick technique. Laryngoscope (2010) 120(5):975–7. doi: 10.1002/lary.20862 20422694

[24] Labidi M Watanabe K Hanakita S Park HH Bouazza S Bernat AL . The chopsticks technique for endoscopic endonasal surgery-improving surgical efficiency and reducing the surgical footprint. World Neurosurg (2018) 117:208–20. doi: 10.1016/j.wneu.2018.05.229 29886295

